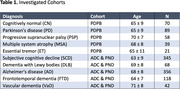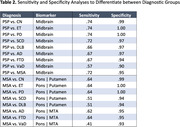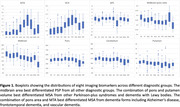# Differentiating Parkinson‐Plus Syndromes from Other Neurodegenerative Diseases Using Automated T1 MRI Quantification

**DOI:** 10.1002/alz.088632

**Published:** 2025-01-09

**Authors:** Elena M Bonke, Juha Koikkalainen, Antti Tolonen, Juhana Hakumäki, Patrizia Mecocci, Steen G. Hasselbalch, Hanneke F.M. Rhodius‐ Meester, Bas Jasperse, Afina Willemina Lemstra, Wiesje M. van der Flier, Frederik Barkhof, Jyrki Lötjönen

**Affiliations:** ^1^ Combinostics Ltd, Tampere Finland; ^2^ University of Eastern Finland and Kuopio University Hospital, Kuopio Finland; ^3^ Institute of Gerontology and Geriatrics, Department of Medicine and Surgery, University of Perugia, Perugia Italy; ^4^ University of Copenhagen, Department of Clinical Medicine, Copenhagen Denmark; ^5^ Vrije Universiteit Amsterdam, Amsterdam UMC, Amsterdam Netherlands; ^6^ UCL Institutes of Neurology and Healthcare Engineering, London UK

## Abstract

**Background:**

Accurate differential diagnosis of neurodegenerative diseases is challenging, but crucial for the management and treatment, particularly given the development of disease‐modifying drug therapies for Alzheimer’s disease (AD). In this work, we investigate imaging biomarkers derived from T1‐weighted magnetic resonance imaging (MRI) with a focus on differentiating Parkinson‐plus syndromes from other relevant diagnostic groups in dementia and Parkinson’s disease (PD).

**Method:**

MR scans from 1206 subjects and three cohorts were used: Parkinson’s Disease Biomarkers Program (PDBP), Amsterdam Dementia Cohort (ADC) and PredictND cohort (PND) (Table 1). The following imaging biomarkers were quantified using an automated image quantification tool: computed medial temporal lobe atrophy score (MTA), computed global cortical atrophy score (GCA) [1], anterior vs. posterior score (APS) [2], the midsagittal areas of the midbrain and pons and midbrain/pons ratio, the volumes of the cerebellum and putamen. Percentiles versus age, sex and head size normalized data were used to calculate sensitivity and specificity. As Parkinson‐plus syndromes are quite rare, a cutoff for each marker was chosen to ensure high specificity and avoid false positives.

**Result:**

Figure 1 shows boxplot visualizations for all investigated imaging biomarkers and diagnostic groups. Table 2 shows the performance in differential diagnosis. 1) The midbrain area best differentiated progressive supranuclear palsy (PSP) from all other diagnostic groups. 2) The combination of pons area and putamen volume best differentiated multiple system atrophy (MSA) from other PD forms. 3) The combinations of pons area and MTA best differentiated MSA from several dementia forms.

**Conclusion:**

Automatic MRI quantification of brain structures such as the midsagittal area of the midbrain and pons, putamen volume and MTA rating can help clinicians to differentiate between common neurodegenerative diseases like AD, vascular dementia and frontotemporal dementia and more Parkinson‐related neurodegenerative diseases like MSA, PSP, and dementia with Lewy bodies.

**References**:

[1] Koikkalainen et al., European Radiology, 2019

[2] Bruun et al, NeuroImage Clinical, 2019